# Obesity and its health impact in Africa: a systematic review

**DOI:** 10.5830/CVJA-2012-040

**Published:** 2012-10

**Authors:** Bridget Adeboye, Giovanna Bermano, Catherine Rolland

**Affiliations:** Centre for Obesity Research and Epidemiology (CORE), Faculty of Health and Social Care, Robert Gordon University, Aberdeen, Scotland; Centre for Obesity Research and Epidemiology (CORE), Faculty of Health and Social Care, Robert Gordon University, Aberdeen, Scotland; Centre for Obesity Research and Epidemiology (CORE), Faculty of Health and Social Care, Robert Gordon University, Aberdeen, Scotland

**Keywords:** obesity, Africa, prevalence, cardiovascular risk

## Abstract

**Abstract:**

Obesity and its association with co-morbidities in Africa are on the rise. This systematic review examines evidence of obesity and its association with co-morbidities within the African continent. Comparative studies conducted in Africa on adults 17 years and older with mean body mass index (BMI) ≥ 28 kg/m^2^ were included. Five electronic databases were searched. Surveys, case–control and cohort studies from January 2000 to July 2010 were evaluated. Of 720 potentially relevant articles, 10 met the inclusion criteria. Prevalence of obesity was higher in urban than rural subjects with significant increases in obesity rates among women. Inflammatory marker levels were significantly elevated among Africans compared with Caucasians. The co-relationship between obesity and chronic diseases was also highlighted. This systematic review demonstrates that while obesity remains an area of significant public health importance to Africans, particularly in urban areas, there is little evidence of proper diagnosis, treatment and/or prevention.

## Abstract

Obesity has long been acknowledged as a significant contributing factor in the development of various chronic diseases such as cardiovascular disease, hypertension, type 2 diabetes mellitus, stroke, osteoarthritis and certain cancers.^1^-^3^ As a risk factor for non-communicable diseases, obesity has become a global public health concern with more than one billion adults estimated to be overweight and over 400 million of them obese.^4^,^5^

Recent global figures from the World Health Organisation (WHO) indicate that the prevalence of obesity is not just a problem of the developed countries but is also on the increase in the developing world, with over 115 million people suffering from obesity-related problems.^5^ This significant acceleration in the incidence of obesity also indicates that low-income countries are now confronted with a double burden where both communicable and chronic non-communicable diseases co-exist.^6^-^8^

While the threat of communicable and poverty-related diseases (such as malaria, malnutrition, cholera and infant mortality) exists in several African countries, the prevalence of chronic diseases continues unabated.^2^,^9^-^12^ In fact, several researchers predict that in many developing countries, the burden of chronic diseases will equal the burden of acute infectious diseases in the near future.^7^,^13^,^14^ For instance, the WHO projects by 2030 a doubling in mortality rates resulting from ischaemic heart disease in the African region,^15^ as well as a prediction by 2025 of the largest increase in prevalence of diabetes mellitus in developing countries.^16^

Despite this increasing need to tackle chronic diseases with additional resources and effort,^17^ under-nutrition and communicable infectious diseases remain a core focus of researchers and policy makers within the African continent,^11^,^18^ with insignificant attention assigned to obesity and chronic, non-communicable diseases.^2^,^6^ The lack of attention given to the obesity epidemic may have been spurred on by the earlier misrepresentation of health information, which led to the misperception of ‘healthy’ or ‘benign’ obesity.^19^,^20^

This concept, widely propagated in places such as South Africa from the 1960s until the late 1980s,^20^,^21^ led to gross neglect of the problem of obesity and treatment of the attendant co-morbidities.^19^,^20^ Although the misperception of ‘benign obesity’ is being rectified by the increasing number of recent studies that spell out the reality of obesity,^22^,^23^ the threat of diseases such as HIV/AIDS and the high economic toll it takes on the continent make it increasingly difficult to divert resources to tackling the obesity epidemic.^24^-^26^

This systematic review focuses on epidemiological studies (surveys, case–control and cohort) with comparative subgroups. It aimed to unearth the current evidence on obesity and its association with increased co-morbidities among obese individuals on the African continent. The pattern of obesity in Africa was explored, comparing the differences in prevalence between urban and rural subjects. General outcome, such as prevalence of obesity among urban residents, was highlighted to show the impact of urbanisation and Westernisation. The review also highlights the impact of obesity on cardiovascular and inflammatory bio-markers, comparing Africans and Caucasians. Co-morbidities of obesity and their prevalence among the obese in comparison with the non-obese population were also evaluated.

## Methods

A comprehensive electronic literature search of five databases (Cochrane library, Medline, EMBASE, CINAHL and Amed) was conducted using both medical subject headings (MeSH) and key text such as ‘obesity’, ‘overweight’, and ‘BMI’. Using the appropriate Boolean operators, key search words were combined with Africa, exploding searches to include West Africa, East Africa, Africa sub-Sahara, South Africa, Central Africa and North Africa. The search was restricted to studies on human subjects published between January 2000 and July 2010. Language restrictions were applied, limiting searches to publications in only the English language.

The eligibility criteria for inclusion were: randomised control, epidemiological, case-controlled, cohort studies, and surveys with a mean body mass index (BMI) of ≥ 28 kg/m^2^ involving participants aged 17 years and older. The rationale for a lower BMI cut-off point was to allow the consideration of co-morbidities. Moreover, at that level of body fat and age, individuals of African descent have been shown to have a lower BMI compared to that of Caucasians.^27^ Studies also had to be conducted in an African country to be included, and had to have a comparative aspect (urban vs rural, African-based population vs Westernised counterpart, gender, etc).

Titles and abstracts were screened and potentially relevant articles were retrieved. Reference lists of review articles were searched manually and a few eligible articles were retrieved. Full texts of potential articles were retrieved and examined for inclusion by two reviewers.

Data from each eligible study were extracted based on a standard protocol format recommended by the Cochrane collaboration.^28^,^29^ The data-extraction form was adapted for this review after piloting a few studies and making alterations where necessary, to ensure standardisation prior to final data extraction. Data were extracted by one reviewer and independently reviewed by a second reviewer. Uncertainties and discrepancies were rectified and resolved by discussion with the two reviewers.

## Results

**Fig. 1.**
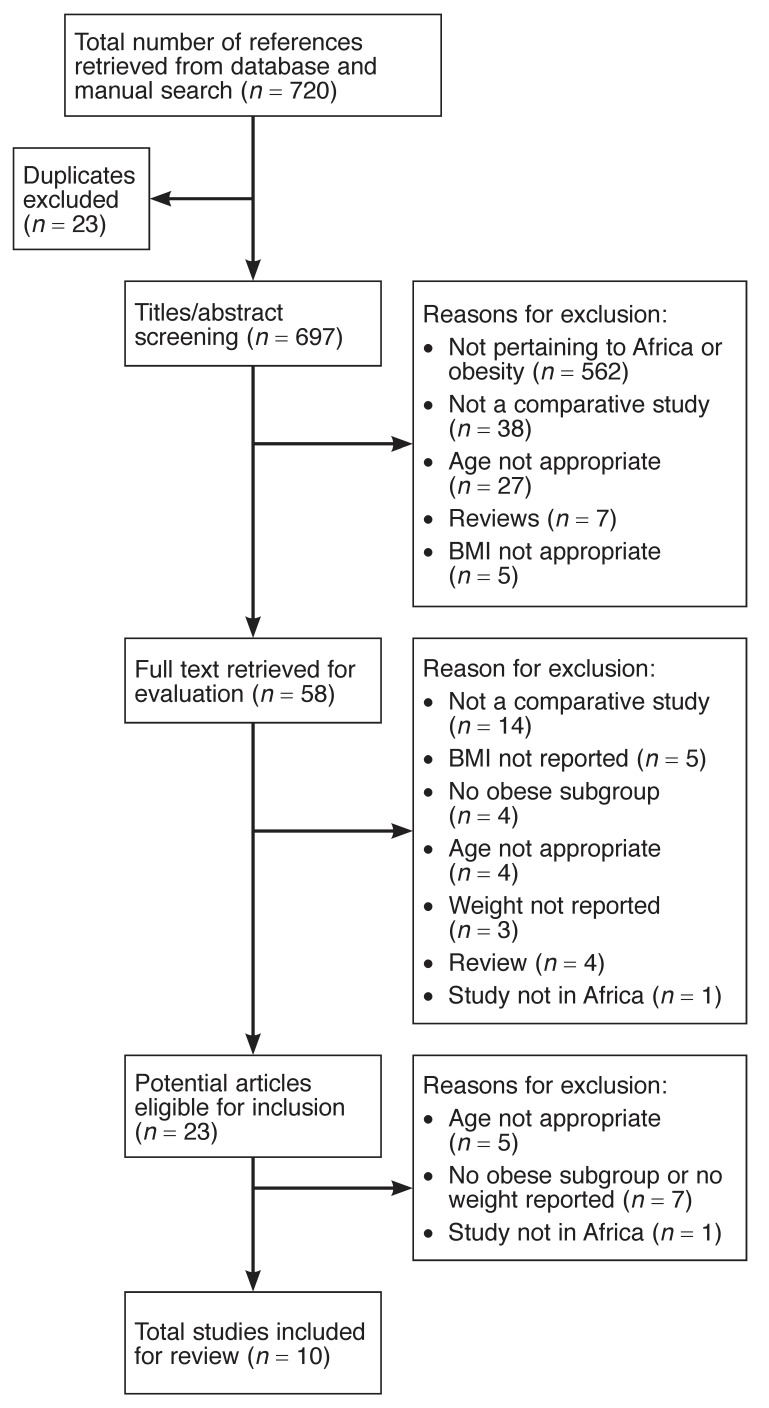
Summary of literature search.

The initial search generated 720 titles of potentially relevant articles. Further scanning of titles and abstracts yielded 58 potentially relevant articles for which full texts were obtained. A total of 10 articles met the inclusion criteria and were included in the review. Fig. 1 outlines a summary of the selection process with reasons for the exclusions.

All the included studies had comparative subgroups. Of the 10 studies included (Table 1), two were observational,^30^,^31^ three cross-sectional;^32^-^34^ three case–case controlled studies,^35^-^37^ and two were surveys.^2^,^38^

**Table 1.** Characteristics And Contextual Details Of All The Included Studies

*Study*	*Sample size total (women)*	*Study design*	*Country of origin*	*Inclusion criteria*	*Aim of study*	*Outcome measured (comparison evaluated)*
Agyemang *et al*.^34^	1 471 (ND)	Cross sectional	Ghana and Netherlands	Urban and rural adults and their Netherlands counterparts ≥ 17 years	To assess the differences in overweight and obesity between Dutch–Ghanaian migrants in Netherlands and their rural/urban counterparts in Ghana.	BMI, obesity (urban vs rural population with their European counterparts (males vs females)
Amoah^33^	4 731 (2 874)	Cross sectional	Ghana	Urban and rural adults ≥ 25 years	To determine the association between obesity and socio-demographic factors in Ghana	BMI, %obesity prevalence (urban vs rural population, males vs females)
Asfaw^2^	3 190 (ND)	Health survey	South Africa and Senegal	Adults in South Africa and Senegal ≥ 18 years	The effects of obesity on doctordiagnosed chronic diseases in Africa	BMI, age, doctor-diagnosed comorbidities (obese vs non-obese population)
Fezeu *et al*.^10^	3 160 (ND)	Cross sectional	Cameroon	Urban and rural adults ≥ 24 years	To compare the 10-year changes in the distribution of adiposity in rural vs urban Cameroonian population	BMI, WC (urban vs rural population, males vs females)
Ibhazehiebo *et al*.^36^	120 (60)	Case–control	Nigeria	18–22 years	To determine the association of obesity with premature increase in BP	BMI, weight, SBP, DBP (obese vs non-obese, males vs females)
Jackson *et al*.^30^	2 855 (ND)	Cross sectional	Cameroon, Jamaica and UK	Age 25–74 years; not pregnant and of African descent by ancestry, observed race and self-assignment	To determine the relationship between diet and obesity	BMI, socio-demographic factors (rural vs urban and Africans in diaspora) with age taken into account
Rush *et al*.^31^	721 (721)	Observational	South Africa and New Zealand	18–60 years	To investigate the relationship between BMI and %BF among 5 ethnic groups	BMI, %BF , WC (South African black vs South African European)
Schutte *et al*.^37^	98 (98)	Case–control	South Africa	Urban adults ≥ 18 years	Determine the relationship between HBP and leptin levels in African women	BMI, weight, leptin level (normotensive vs hypertensive African women)
Schutte *et al*.^35^	217 (217)	Case–case control	South Africa	Urban adults 20–50 years	Relationship between inflammation, obesity and cardiovascular disease.	Cardiovascular and inflammatory bio-markers (SBP, DBP, CO, TRP, leptin, HsCRP and fibrinogen (Africans vs Caucasians)
Schutte *et al*.^32^	217 (217)	Cross sectional	South Africa	Urban adults 20–55 years	To determine the relationship between BMI, HBP and cardiovascular and inflammatory biomarkers	BMI, DBP, SBP, leptin, CRP and hypertension % (Africans vs Caucasians)

ND, not defined; hsCRP, high-sensitivity C-reactive protein; %BF, percentage body fat; WC, waist circumference; BMI, body mass index; SBP, systolic blood pressure; DBP, diastolic blood pressure; HBP, high blood pressure; CO, cardiac output; TPR, total peripheral resistance.

The age of the participants ranged from 17 to 74 years. Six of the included studies were conducted on both males and females,^2^,^10^,^30^,^33^,^34^,^36^ while four were exclusively on female populations.^31^,^32^,^35^,^37^ Sample sizes varied widely, ranging from 98 to 4 731 participants.^33^,^37^

Two studies reported results from rural and urban populations, while two others reported findings from rural and urban populations and compared them with their Western counterparts.^30^,^34^ The study by Rush *et al*.^31^ was the only one conducted within an urban population, comparing BMI and percentage body fat differences between women from five ethnic groups.

Schutte was the first author of three of the studies included.^32^,^35^,^37^ Participants from Schutte’s studies, known as POWIRS (Profiles of Obese Women with Insulin Resistance Syndrome), were all recruited from affluent parts of South Africa. Two of Schutte and co-workers’ studies compared inflammatory and cardiovascular risk markers between two ethnic groups (African women vs Caucasians).^32^,^35^ The third by Schutte *et al.* considered only African women, exploring the association of leptin and BMI between overweight/obese hypertensive (HT) and normotensive (NT) participants.^37^

Ibhazehiebo’s was the only study conducted within an institution of higher learning (medical students in a private university in Nigeria).^36^ Ibhazehiebo compared blood pressure changes between obese and non-obese (control group) participants following graded exercise.^36^ Asfaw’s on the other hand was the only study that compared the effects of obesity on four doctor-diagnosed chronic diseases, reporting results from both Senegal and South Africa.^2^

## Comparison of demographic variables and BMI across groups/gender (crude prevalence of obesity by gender and locality)

Table 2 shows the four studies that reported on demographic variables. The prevalence of obesity varied extensively between and within studies. In all the regions studied, the difference in the prevalence of obesity between males and females was significant, with women as much as three times more likely to be obese than their male counterparts in some regions (22.5 and 5.9%, respectively).^33^

**Table 2.** Prevalence Of Obesity Across Location/Gender

*Author Country*	*Urban subjects*		*Rural subjects*		*Western counterparts*	
	*Men*	*Women*	*Men*	*Women*	*Men*	*Women*
Agyemang *et al*.^34^ (Ghana/Netherlands)	11 (3.0)^a^	71 (17.0)^a^	1 (0.5)	20 (6.3)^b^	13 (19.1)^b^ Dutch Ghanaians	23 (25.9)^b^ Dutch Ghanaians
Amoah^33^ (Ghana)	5.9%	22.5%	2.0%	15.8%	–	–
Fezeu *et al*.^10^ (Cameroon)	28.2%	11.9 %	1.5%	2.1%		
(95% CI)	(24.6–32.1)	(9.1–15.2)	(0.4–3.4)	(1.0–4.0)	–	–
1994 data	27.4%	13.8%	1.8%	7.8%		
2003 data	(23.6–31.5)	(10.6–17.7)	(0.4–5.0)	(4.2–12.4)	–	–
Jackson *et al*.^30^ (Cameroon and UK)	27.0 (5.0) (25.2%)	25.0 (3.6) (10.0%)	22.3 (3.3) (3.3%)	21.7 (2.6) (0.7 %)	28.6 (5.7) (37.1 %)	27.3 (3.5)^a^ (21.6%)

Data are presented as means and standard deviations in brackets unless stated otherwise. ^a^p < 0.001 significant difference between groups and between genders (the degree of difference is the same across a and b) ^b^p < 0.001 statistically significant comparing urban to rural counterpart. 95% CI: 95% confidence interval.

When studies on urban and rural populations were analysed, the prevalence of obesity was found to be higher in the urban than the rural population (Table 2).^33^,^34^ One study from Ghana investigated the differences in overweight and obesity between rural and urban Ghanaians and compared them with first-generation Dutch–Ghanaian migrants in the Netherlands.^34^ Findings showed the prevalence to be lowest for both males and females in the rural regions and highest among their Western counterparts.

A similar trend was observed in a study by Jackson *et al*.,^30^ which examined overweight and obesity among populations of African origin in Cameroon, Jamaica and the UK. They reported that levels of overweight and obesity were higher in those who had migrated to the UK than those who lived in Cameroon or Jamaica. Compared to other sites, obesity was found to be at its lowest level in rural males and females in Cameroon. In fact, due to the rarity of obesity in rural Cameroon, the site was omitted from the analyses of obesity. Rural Cameroon was used however as the reference category for analyses of overweight, and urban Cameroon for analyses of obesity.^30^

Two other studies reported on the age- and gender-specific prevalence of obesity.^33^,^34^ In all localities, the prevalence of overweight/obesity among men was higher in the older age group than the younger age group. In addition, a significant prevalence was observed in younger and older Dutch–Ghanaian men (50.0 and 84.2%) compared with their urban (14.1 and 39.2%) and rural Ghanaian counterparts (5.6 and 16.7%). A higher prevalence of overweight and obesity was also evident among younger and older Dutch–Ghanaian women (65.0 and 94.7%) compared with their urban (44.5 and 61.0%) and rural Ghanaian counterparts (17.8 and 28.4%).^34^

In one study,^33^ the most significant prevalence of obesity in both genders occurred between the ages of 55 and 65 years, at 7.8 and 32.9% in men and women, respectively.^33^ In another study,^30^ middle-aged urban men were found to be more prone to becoming obese than younger men. However, only risk of obesity and not overweight was evident among older men (60–74 years) compared with younger men.

Similarly, women within the same age group (41 years and older) in urban Cameroon were also found to be at increased risk of developing obesity. When compared with younger men, the risk of overweight and obesity increased among men aged 41 to 59 years.

When age was adjusted for, the rural population in Cameroon were excluded from the analysis because of non-significant results. However, at age 41 to 59 years, there was a significant increase in obesity across the two geographic areas (urban Cameroon and the UK), which began to decline from age 60 to 70 years. Data from Jamaican subjects were excluded in this review as they were not considered to be European counterparts.^30^

## Comparison of cardiovascular parameters and inflammatory markers

Three studies by Schutte *et al.* investigated the association between cardiovascular and inflammatory bio-markers with obesity (Table 3).^32^,^35^,^37^ They were part of the POWIRS study and were carried out in South Africa by the same group.

**Table 3.** Comparison Of Cardiovascular Parameters And Inflammatory Bio-Markers Across Ethnic Groups

*Author*	*Control vs experimental groups*	*Outcome measured (cardiovascular parameters and inflammatory bio-markers)*				
		*SBP (mmHg)*	*DBP (mmHg)*	*Leptin (ng/ml)*	*hsCRP (mg/l)*	*Fibrinogen (g/l)*
Schutte *et al*.^28^	OW/OB NT (*n* = 46)	124 ± 1.9	77 ± 1.2	73.6 ± 3.4	–	–
	OW/OB HT (*n* = 17)	156 ± 1.9^a^	91 ± 2.1^a^	69.8 ± 5.7	–	–
Schutte *et al*.^26^	Caucasians (*n* = 115)	125 (123; 128)	72.5 (70.8; 74.1)	51.4 (45.3; 57.5)	3.27 (2.56; 3.98)	3.05 (2.95; 3.15)
	Africans (*n* = 102)	130 (126; 134)^a^	77.7 (75.6; 79.8)^b^	57.6 (51.6; 63.6)^a^	4.59 (3.17; 6.01)	3.89 (3.67; 4.10)^b^
Schutte *et al*.^23^	Caucasians (*n* = 115)	119 ± 12.1	74.3±8.78	51.4 ± 32.9	3.27 ± 3.84	3.05 ± 0.56
	Africans (*n* = 102)	128 ± 20.3^a^	78.5±12.0	57.6 ± 30. 2^a^	4.59 ± 7.20	3.89 ± 1.08^a^

All three studies took place in South Africa. OW/OB NT, overweight/obese normotensive; HT, hypertensive; SBP, systolic blood pressure; DBP, diastolic blood pressure; hsCRP, high-sensitivity C-reactive protein. Schutte *et al*. 2005 results report mean ± (standard deviation); Schutte *et al*. 2006 values are mean ± (95% confidence intervals); Schutte et al. 2008 values are mean ± (standard deviation). ^a^p < 0.05 when comparing the control versus the experimental group ^b^p < 0.001 when comparing the control versus the experimental group

With the exception of the study by Schutte *et al*.,^37^ which compared normotensive and hypertensive African women, the remaining two studies investigated the differences in response between African and Caucasian women.^32^,^35^ Schutte and co-workers’ findings showed a significant elevation of leptin levels (*p* < 0.05) in the overweight and obese normotensive (OW/OB NT) and hypertensive (HT) groups in comparison with the lean NT group, but it was similar in the OW/OB NT and HT groups.^37^

Matching healthy African (*n* = 102) and Caucasian (*n* = 115) women for age and BMI, Schutte *et al.*^35^ sought to determine the role of ethnicity. In their investigation of the relationship between inflammation, obesity and cardiovascular disease in a South African population, they found significantly increased levels of leptin, high-sensitivity C-reactive protein (hsCRP) and fibrinogen (*p* < 0.05) in the African women compared with their Caucasian counterparts (Table 3).

Similarly, Schutte *et al*.^32^ investigated the differences in blood pressure (BP) for age- and BMI-matched African women and their Caucasian counterparts. Their study sought to determine whether obesity was strongly connected to reported cardiovascular risk markers in black African women. The results revealed that although the mean BMI and age were matched between the two groups, the Caucasians were significantly taller (1.68 ± 0.07 vs 1.59 ± 0.06 m; *p* < 0.01) and heavier (80.7 ± 21.0 vs 70.6 ± 15.8 kg; *p* < 0.01). Moreover, the African women had higher systolic blood pressure than the Caucasians (128 ± 20.3 and 119 ± 12.1 mmHg, respectively) (*p* < 0.01) with higher peripheral vascular resistance.

## Comparison of the effects of obesity on blood pressure and doctor-diagnosed chronic diseases

Ibhazehiebo and colleagues’ case–control study reported hypertension and blood pressure responses to graded exercise in young obese and non-athletic Nigerian university students (Table 4).^36^ By contrast, Asfaw’s study considered the impact of obesity on the prevalence of chronic diseases (four doctordiagnosed chronic diseases) in South Africa and Senegal (Table 5).^2^ Although these two studies were not comparable, both their findings demonstrated a greater incidence of disease in the obese than in their non-obese counterparts.

**Table 4.** Effects Of Obesity On Blood Pressure Following Graded Exercise In Obese And Non-Obese Subjects^27^

*Author, country*	*Sample size*		*Degree of exercise*	*Exercise Blood pressure changes following graded exercise*			
	*Males*	*Females*		*SBP (mmHg) obese*	*SBP (mmHg) non-obese*	*DBP(mmHg) obese*	*DBP (mmHg) non-obese*
Ibhazehiebo *et al*.^36^	60	60	Mild (M)	156.3 ± 14.0	135.0 ± 7.4	87.9 ± 10.0	70.1 ± 7.4
Nigeria			Mild (F)	141.0 ± 9.0^a^	134.5 ± 10.5	86.4 ± 10.1	74.6 ± 4.2
			Mild overall	150.4 ± 10.3^b^	94.3 ± 8.6	84.5 ± 8.6	78.3 ± 9.4
			Moderate (M)	163.4 ± 10	148.2 ± 14.8	93.6 ± 7.0	84.6 ± 12.4
			Moderate (F)	152.3 ± 11.0^a^	140.3 ± 11.6	92.0 ± 6.0	78.3 ± 9.2
			Moderate overall	161.7 ± 9.6 ^b^	113.8 ± 10.1	91.7 ± 6.0	83.9 ± I7.5
			Severe (M)	173.1 ± 14.0	153.0 ± 13.1	98.5 ± 10.0	89.2 ± 11.5
			Severe (F)	163.1 ± 9.0^a^	148.8 ± 11.8	95.4 ± 10.0	85.8 ± 10.9
			Severe overall	169.4 ± 11.2^b^	126.0 ± 10.8	97.4 ± 8.9	89.3 ± 9.2

SBP, systolic blood pressure; DBP, diastolic blood pressure (mmHg); M, males; F, females; mean ± (standard deviation) ^a^p < 0.05 between genders. ^b^p < 0.001 between obese and non-obese groups.

**Table 5.** Prevalence Of Chronic Disease Among Obese And Non-Obese Individuals From Two Different Countries^2^

*Author, country*	*Sample size total*	*Doctor-diagnosed chronic diseases*	*Obesity status*	*Senegal*	*South Africa*
Asfaw 2	3 190	Arthritis	Non-obese	17.3 (15.4–19.2)	17.1 (14.1–20.0)
South Africa/Senegal			Obese	24.1 (15.8–32.3)	22.4 (16.9–27.1)
		Asthma	Non-obese	4.2 (3.2–5.2)	5.4 (4.1–6.9)
			Obese	10.0 (4.1–15.9)	7.6 (5.3–10.5)
		Diabetes	Non-obese	1.6 (1.0–2.2)	5.4 (4.1–6.8)
			Obese	2.9 (0.4–6.2)	7.6 (5.1–10.2)
		Heart disease	Non-obese	7.8 (6.7–9.1)	13.3 (11.3–15.3)
			Obese	13.7 (7.0–20.4)	19.4 (15.7–23.3)

Values are mean (95% confidence interval); significant differences were not clearly reported

Results from Ibhazehiebo *et al*.^36^ revealed a significant (*p* < 0.001) increase in the incidence of hypertension among the obese participants, with no incidence of hypertension recorded in the non-obese group (Table 4). Ibhazehiebo also found a considerable increase (*p*-value not reported) in the systolic (SBP) and diastolic blood pressure (DBP) of the obese subjects following graded exercise. The increase heightened as the intensity of the exercise progressed from mild to severe (Table 4). By contrast, only modest increases were observed in the SBP and DBP values of the non-obese subjects. When increases in BP were compared in both groups following graded exercise, highly significant increases in SBP were observed in the obese groups (*p* < 0.01) (Table 4).

Asfaw’s study displayed similar trends, suggesting a clear relationship between obesity and four diagnosed chronic diseases (arthritis, asthma, diabetes and heart diseases).^2^ When compared with non-obese subjects, each chronic disease was more prevalent in the obese respondents (Table 5).

## Discussion

We systematically reviewed the available literature in this article to assess current evidence on obesity and its association with increased co-morbidities among obese individuals on the African continent. The results of this review demonstrate a higher prevalence of obesity in urban populations compared with their rural counterparts. Studies from both Amoah^33^ and Agyemang *et al*.^34^ reported similar findings in their urban and rural populations, however, it was intriguing to see Agyemang *et al*.^34^ compare rural and urban Ghanaians with their Dutch counterparts.

The large disparity found between Ghanaian residents in the Netherlands and their urban/rural counterparts suggests that environmental factors are implicated in the aetiology of obesity.^34^ This provides new insights into the possible role of migration-related factors on overweight and obesity in Western countries. This further signals a great need to address overweight and obesity among migrant populations living in Western countries. It also provides an essential bedrock for further studies to ascertain migration-related lifestyle changes and factors that lead to overweight and obesity among these populations in Western countries.

Studies reporting a higher prevalence of overweight and obesity among urban residents are consistent with recent studies in African countries;^6^,^38^,^39^-^41^ however, the results from the studies presented in this review seem to be at variance with most urban–rural analyses of obesity in some developed countries.^42^ For instance, in the UK and USA, some studies reported a higher proportion of obesity and overweight in the rural than the urban population.^30^,^43^ Higher prevalence of obesity was also found in those with a lower income and least education in developed countries,^44^,^45^ contradicting the findings in this review that associated obesity with affluence and literacy among African populations.

Most observational studies conducted in industrialised countries also suggested a positive association of obesity with low-income and deprived neighbourhoods.^46^-^50^ This obvious contradiction has been suggested to be due to heightened deprivation^42^ and exposure to poor-quality foods, which are the default choice due to low income^42^ among the poor in Western countries.

On the contrary, a possible explanation for the higher prevalence of obesity in affluent and urban populations in Africa may be explained by the increasing evolvement of urbanisation within the African continent. The majority of African countries are undergoing swift changes in their social and economic environments, concommitant with changes in food-consumption patterns.^51^ The increasing availability of food and its diversity in urban areas has been shown to particularly influence the quality of diets and nutritional well-being.^52^ These changes in diet and lifestyle, especially in urban settings, often involve a shift from the consumption of traditional staple foods low in fat and rich in fibre, to processed and refined foods, meat and dairy products, high in saturated fats and sugar.^52^-^54^

The consequence of urbanisation, which is often connected with the adoption of a lifestyle commonly referred to as ‘westernisation’, is the increased intake of energy-dense foods and high-calorie sugary meals and drinks.^55^-^57^ Urbanisation is also associated with less energy-demanding jobs, complemented by increased sedentary lifestyles and the adoption of detrimental eating habits, which include the regular consumption of fast food and so called ‘eat out’.^55^-^57^

With increasing economic development and urbanisation comes the proliferation of fast-food chains, and easy and cheaper means of transport, which are mostly patronised by the affluent. All of these factors above may have contributed to the higher prevalence of obesity noticed in the urban and affluent populations of African countries. Changes in lifestyle are therefore convincingly implicated as causative factors in the observed higher prevalence of obesity in urban than in rural populations.

Existing studies have also implicated urbanisation in the increasing burden of co-morbidities of obesity such as cardiovascular disease (CVD), type 2 diabetes and hypertension. For instance, the study by Niakara and co-workers in a West African urban environment revealed a high incidence of hypertension (40.2% in a sample of 2 087 participants).^58^ A similar study by Sobngwi *et al*.^59^ also associated urbanisation and socio-economic factors as driving forces in the increasing prevalence of hypertension in West Africa.^59^

Similarly, Amoah^33^ and Jackson *et al*.^30^ reported a positive association of age with overweight and obesity. They identified middle-aged respondents as having the highest prevalence of obesity, which began to decline from the age of 65 years. This links increasing age with the development of obesity, a factor that should be considered by policy makers when addressing obesity in the African continent. The decline in prevalence of obesity in older age has also been noticed in other parts of the world.^60^,^61^

The results of this review also suggest a higher prevalence of obesity in the female population compared to their male counterparts. This higher prevalence in women depicts the global situation in most populations of African origin, including those in the diaspora.^34^,^62^,^63^ Although there is no direct comparison, about 77% of African women in the diaspora are reportedly overweight or obese, typifying the female gender as the most obese population compared with their male counterparts as well as with males of all other ethnic groups.^43^,^61^

Preferred body image may be a key factor in obesity among African women.^64^ Numerous studies suggest a preference for overweight over normal BMI among African women.^65^,^66^ Moreover, cultural perceptions concerning obesity and overweight may also have played a role in the high prevalence of overweight and obesity among women, and central obesity among affluent men in this review.

Culture is known to shape health behaviour and serves as a mirror for perceiving and interpreting experiences.^67^,^68^ In several parts of Africa, obesity is currently held with little opprobrium,^69^,^70^ people generally associating fatness with beauty, fame and evidence of good living and health,^33^,^65^,^71^ particularly in women.^72^ In some cases, women are fattened up for suitors prior to marriage,^73^,^74^ as a sign of beauty and fertility. Furthermore, African men are purported to have a preference for overweight over thin women.^33^

Although affluence, effects of urbanisation and excess consumption of food are mainly implicated in the increased prevalence of obesity on the African continent (as suggested by this review), food insecurity may also be a factor to consider at the other end of socio-economic spectrum.^64^ Townsend and colleagues^75^ suggested a positive association of food insecurity with overweight in women, while Chaput *et al*.^76^ linked food insecurity to overweight status in women but not in men in Uganda. Conclusively, women of all socio-economic strata in the African continent can be said to be at risk of developing overweight and obesity, albeit through diverse mechanisms, which may necessitate further research.

From all three studies comparing cardiovascular and inflammatory bio-markers, results showed that the levels of leptin, hsCRP and fibrinogen were significantly higher in African women. African women also had higher blood pressures and vascular resistance than their Caucasian counterparts. However this condition may have been induced by different factors, such as sodium sensitivity, which is an important condition in the African population.^77^ It is also notable that, although black South Africans had higher blood pressures, vascular resistance, and fibrinogen and leptin levels than their Caucasian counterparts, the relationships of these markers with obesity were markedly weaker than those of Caucasians, suggesting that the mechanisms to elucidate the weaker correlations of cardiovascular indices and obesity in the African population remain unresolved.^32^

The above results also imply that the level of clinical markers should not necessarily be compared between ethnic groups, as the effectiveness and sensitivity of a specific biochemical marker might be completely different in an ethnically distinct group. Findings further raise the question whether obesity should be regarded as a cardiovascular threat to these women. By contrast, given that the obesity measures of African women were strongly linked with markers that are more associated with type 2 diabetes, such as triacylglycerols, inflammation and insulin resistance, this suggests that obesity in African women may have a primary effect on the development of diabetes, and secondarily on cardiovascular disease.

Earlier studies in South Africa have shown ischaemic heart disease (IHD) to be more prevalent among Europeans,^21^,^78^-^80^ while type 2 diabetes^81^-^83^ and hypertension^84^ were more common among Africans. Although the metabolic reasons behind these differences remain complex, more recent studies suggest that black South Africans have a less atherogenic fasting lipid profile than their white counterparts.^85^-^87^ Crowther *et al*.^87^ also observed greater amounts of visceral fat and waist-to-hip ratios in obese whites than in obese black individuals.^87^

Given that the study by Schutte *et al*.^32^ did not include environmental influences in its investigation of these relationships, caution should be applied in the interpretation of these results. The seasonal variation in the collection of data and assay dates between the two ethnic groups could have induced bias and may have influenced the resulting differences found in the levels of inflammatory markers.

Another consideration in the interpretation of the results is the age of the women in this study, as they were relatively young (mean age 31.3 years). Although their inflammatory markers were elevated due to obesity, it is likely that established atherosclerosis and vascular dysfunction (and related inflammatory conditions) were non-existent in this group.^32^

Other results from this review also suggest an association of obesity with a premature increase in blood pressure. A considerable number of obese young adults in the study by Ibhazehiebo *et al*.^36^ were hypertensive compared with the non-obese control group. Marked increases in SBP were also observed in the obese individuals compared to the controls at all levels of graded exercise, with the highest values seen during extreme exercise. These findings suggest that obese young individuals were prone to early onset of hypertension, a situation that makes them susceptible to cardiovascular complications and other health problems in the future.

The findings of Ibhazehiebo *et al*.^36^ were also consistent with several other studies that associated increased BMI with increased risk of hypertension. The study by Wolf *et al*.^88^ found that the risk of hypertension was up to five times higher among obese people than in their normal-weight counterparts.^88^ Obesity was also positively associated with type 2 diabetes,^6^,^89^,^90^ and it was noted that nearly 90% of individuals who progressed to type 2 diabetes had BMIs above 23.0 kg/m^2^. The compelling association between obesity, hypertension and diabetes among populations of African descent has also been documented.^91^-^93^

Asfaw’s comparison of the prevalence of diagnosed chronic diseases between obese and non-obese subjects also provides an indication of the effect of obesity on the prevalence of chronic diseases.^2^ Each chronic disease included in the study was more prevalent in obese respondents than in their non-obese counterparts. In both countries (South Africa and Senegal), the results indicated that obese subjects were more likely to be diagnosed with two or more chronic diseases than their lean counterparts.^2^ Asfaw’s findings are congruent with numerous studies conducted in both developed and low-income countries.^14^,^94^-^97^

## Limitations

As with many other reviews, this study has a number of limitations, hence the need to apply caution in the interpretation of its findings. Despite the fact that this review sought to incorporate studies from all African countries, all the studies retrieved were articles published in English and from English-speaking countries, reflecting a language bias. It is noteworthy that most studies from African countries are likely to be published in non-indexed and non-English journals.^98^ Moreover, the multilingual, multicultural, multi-ethnic and racial divides that characterise the African continent make it difficult to generalise the findings of this study.

A further limitation was the limited number of studies that fitted the inclusion criteria. Initially, the protocol for this systematic review was designed to evaluate the impact of obesity on the health of participants on the African continent, focusing mainly on ramdomised, controlled trials (RCTs). However, due to the paucity of RCTs, the reviewers settled for comparative studies. This resulted in the omission of some key articles reporting on the extent of the obesity epidemic in Africa.^99^,^100^

Although the comparative aspect of this review is unusual and it resulted in variable data, it did allow us to touch on a broader spectrum of the effects of obesity (from epidemiological to clinical) in Africa. This would not have been feasible if purely epidemiological studies had been included.

Also, the inclusion of three non-population based studies from the same author^32^,^35^,^37^ posed a limitation on the interpretation of the findings in this review. Although the articles met the inclusion criteria of comparative studies, conducting such studies with a larger sample size would have provided more insight into the differences observed in these groups. This highlights the need for larger and more numerous studies of this kind.

Finally, due to the limited number of articles that met the inclusion criteria, as well as the considerable heterogeneity, it is difficult to justify these data as being an accurate representation for the whole of Africa. Hence the need for more specific and structured research on obesity on the African continent.

## Conclusion

The key findings of this review are: (1) obesity was more prevalent in urban than rural areas, particularly in women; (2) obesity was more prevalent in Africans who migrated to Western countries than in their counterparts on the African continent; (3) there was a co-relationship between obesity and chronic disease. However, inflammatory marker levels differed between black and Caucasian individuals and therefore should not necessarily be compared between ethnic groups.

These findings have important public health implications and call for immediate action to combat the increasing prevalence of obesity. This may require a policy shift towards organised and co-ordinated strategies geared at both prevention and treatment of existing obesity.

Provision of facilities that promote physical activities within communities, residential areas and workplaces is essential. Moreover, negative social pressures, cultural perceptions associated with obesity such as ‘fat is beautiful or prosperous’ should be dispelled through educational programmes.^33^ Creation of awareness on individual and community levels of the associated health risks of obesity, augmented by population-based health-promotion programmes are needed. These will highlight the importance of physical activity and healthy eating habits, which will constitute an integral part of forestalling the obesity epidemic and managing its sequelae where it already exists.

Uncurbed, the direct and indirect burden of obesity will be a severe challenge to the future development of Africa as a continent, as well as other developing countries. Neglecting to immediately deal with the problem of obesity and leaving it unchecked will impose additional burdens on the economy and health sector of the African continent, as well as threaten its future development.
